# Cellulose-Silica Nanocomposites of High Reinforcing Content with Fungi Decay Resistance by One-Pot Synthesis

**DOI:** 10.3390/ma11040575

**Published:** 2018-04-09

**Authors:** M. Concepción Rodríguez-Robledo, M. Azucena González-Lozano, Patricia Ponce-Peña, Patricia Quintana Owen, Miguel Angel Aguilar-González, Georgina Nieto-Castañeda, Elva Bazán-Mora, Rubén López-Martínez, Guillermo Ramírez-Galicia, Martha Poisot

**Affiliations:** 1 Universidad del Papaloapan, Circuito Central 200, Parque Industrial, Tuxtepec 68301, Mexico; mc.r.robledo@gmail.com (M.C.R.-R.); memorgal@gmail.com (G.R.-G.); 2 Universidad Juárez del Estado de Durango, Facultad de Ciencias Químicas. Av. Veterinaria S/N, Circuito Universitario, Durango 34120, Mexico; magl62001@yahoo.com.mx (M.A.G.-L.); pattyponcep@yahoo.com (P.P.-P.); 3 Laboratorio Nacional de Nano y Biomateriales, Depto. de Física Aplicada, CINVESTAV Mérida, km 6 Ant. Carr. a Progreso, Mérida 97310, Mexico; p-quint@cinvestav.mx; 4 Facultad de Ciencias, Universidad Nacional Autónoma de México, Circuito Interior, Ciudad Universitaria, CdMx 04510, Mexico; hermescurtus@ciencias.unam.mx (M.A.A.-G.); georginanieto@hotmail.com (G.N.-C.); 5 Departamento de Microbiología y Parasitología, Facultad de Medicina, Universidad Nacional Autónoma de México, CdMx 04510, Mexico; elvabm@gmail.com (E.B.-M.); rlm@unam.mx (R.L.-M.)

**Keywords:** nanocomposites, hybrid materials, one-pot synthesis, thermal stability, fungi decay resistance

## Abstract

Hybrid bionanocomposites based on cellulose matrix, with silica nanoparticles as reinforcers, were prepared by one-pot synthesis of cellulose surface modified by solvent exchange method to keep the biopolymer net void for hosting inorganic nanoparticles. Neither expensive inorganic-particle precursors nor crosslinker agents or catalysts were used for effective dispersion of reinforcer concentration up to 50 wt %. Scanning electron microscopy of the nanocomposites shows homogeneous dispersion of reinforcers in the surface modified cellulose matrix. The FTIR spectra demonstrated the cellulose features even at 50 weight percent content of silica nanoparticles. Such a high content of silica provides high thermal stability to composites, as seen by TGA-DSC. The fungi decay resistance to *Trametes versicolor* was measured by standard test showing good resistance even with no addition of antifungal agents. This one-pot synthesis of biobased hybrid materials represents an excellent way for industrial production of high performance materials, with a high content of inorganic nanoparticles, for a wide variety of applications.

## 1. Introduction

A composite material is obtained by the combination of two or more chemically distinguishable initial components that shows a significant proportion of the raw materials’ properties, thereby delivering a better combination of properties according to the principle of combined action. The continuous phase is called the matrix, while the discontinuous one is known as the reinforcer [1]. The matrix material can be metal, ceramic or polymer, while the reinforcer can be a particle, fiber or sheet [2].

Among sustainable materials, natural fiber composites have such advantages as low cost, light weight, comparable specific strength and stiffness to traditional fiber composites, being renewable and possessing formability with low investment, as well as being environmental friendly [3].

Cellulose fibers have been commonly used for reinforcement of polymer matrix composites that must face several incompatibility problems in order to design an optimal material [4,5].

A vital role that determines such composites performance is the interfacial bonding between fiber and matrix. Usually, the plant-based fiber composite shows limited interaction between the hydrophylic fibers and matrices of common hydrophobic nature that allows poor interfacial bonding affecting the mechanical performance negatively and reducing moisture resistance that drives long term properties [6].

The interfacial bonding can be achieved in several ways, including mechanical interlocking, electrostatic bonding, inter-diffusion bonding and chemical bonding [7,8]. The latter is of particular importance since it delivers strong improvement of composite performance and such a strategy can be applied to the fiber or the matrix. The chemical approach includes treatments of alkali, acetyl, silane, benzyl, acryl, permanganate, peroxide, isocyanate, titanate, zirconate and acrylonitrile reactions plus maleated anhydride grafted coupling agent, but enzyme treatments are becoming more popular due to environmental concerns [9,10].

Several aerogels of cellulose–silica nanocomposites were prepared by sol-gel method application. The in situ formation of silica in the cellulose gel was obtained with tetraethyl orthosilicate (TEOS) precursor and drying with supercritical CO_2_ was required for conversion of composite to aerogel [11]. While another working group approach to prepare aerogels involved methoxytrimethylsilane (MTMS) as silica precursor, the aerogel was obtained by using a freeze dryer [12]. Both reports are very successful but also very energy demanding and time consuming, since several days are required to reach the product. Another group prepared organic–inorganic hybrids of cellulose–silica incorporated with polyhedral oligomeric silsesquioxanes (POSS) by using γ-aminopropyltriethoxy silane (γ-APTES) as a crosslinking agent. Even though the POSS amine particles are well dispersed at the nanometer scale throughout the cellulose matrix, this method is costly [13].

In order to obtain composite materials by an accessible and low-cost method, we have adapted the percolating approach of the solvent exchange method applied to nanofibers of cellulose that involve first forming a three-dimensional template through a self-assembly of the fibers, then filling the percolating architecture with a selected polymer. Before this method, it was impossible to incorporate cellulose particles into nonpolar polymers without the use of surfactants or chemical modifications [14,15].

This work strategy has been employed to avoid using crosslinkers agents and catalysts in order to incorporate the inorganic nanoparticles into the matrix; instead, we used the percolating approach to prepare the surface of cellulose fibers by the solvent-exchange method for effective facial interaction with inorganic particles of hydrophobic behavior, such as nanosilica, to design a new hybrid material of homogenous composition. The main feature of this work is to produce nanocomposites using cellulose as the matrix and not as the reinforcer of any other polymer, with no chemical modifications for interacting with inorganic nanoparticles, following a very facile and cost-effective method. This innovative method significantly reduces the complete synthesis time of the cellulose matrix composites from several days to only a few hours, since it comprises only one step of facile cellulose surface modification. On the other hand, the energy consumed during synthesis is very low and cost-effective, delivering a *Trametes versicolor* resistance cellulose-based hybrid composite with no antifungal agent added, thereby demonstrating low moisture absorption and high dimensional stability, that also shows improved thermal stability against pristine cellulose.

## 2. Materials and Methods 

The nanocomposites synthesis requires the starting precursor materials: silicon dioxide of Aerosil 200, 12 nm particle size with 200 m^2^/g surface area from EVONIK named ASi and silicon dioxide of 100 nm from AVAN-nanoSil named NSi; the cellulose fibers of 20 micron, named C20, were provided by Aldrich (Saint Louis, MO, USA). The first solvent exchange step was adding water in droplets to the cellulose fibers and gently stirring for 15 min to get a gel, then ethanol was added in droplets at 1:1 volume ratio. Stirring was maintained more than 1 h, and then the second solvent exchange step was applied, adding acetone in droplets at 1:2 volume ratio according to water volume. At this point, the surface modified cellulose by solvent exchange method can be dried and named AG20, while the as-received cellulose is named C20. Stirring was still maintained for 3 h more; nanosilica, previously wet with acetone, was added while stirring for 10 min more and 20 min of an ultrasound bath of 40 kHz was also applied, taking care that the temperature did not exceed 40 °C. Finally, the resultant dough was dried at 60 °C in an oven, in order to obtain a fine powder.

The first 8 composite samples were synthesized according to the amounts indicated in Table 1 in order to determine the highest amount of nanosilica that can be uniformly dispersed into the two kinds of matrix prepared: C20 and AG20. There are two main parameters that distinguish these composites; one concerns the kind of matrix: CP1, CP2, CP11 and CP12 contain C20 while CP3, CP4, CP13 and CP14 contain AG20. The other parameter is the amount of NSi increased from 25 to 100 in steps of 25 wt % for every matrix set, giving place to 8 samples in total. In order to determine which one of these matrices can rightly disperse the nanoparticles up to 100 wt %, 4 more samples were synthesized according to Table 1, using only the silica nanoparticles of 12 nm, ASi, in 75 and 100 wt %, comparing the matrix of C20 in CP19 and CP20 and the matrix of AG20 in CP21 and CP22, in order to observe the influence of such silica nanoparticles on the macro and microstructure and properties of the final composites.

The FTIR spectra (Attenuated Total Reflectance mode) were recorded using a Perkin Elmer Spectrum (Akron, OH, USA) 100 with 16 scans per sample. The differential scanning calorimetry (DSC) and thermogravimetric analysis (TGA) were performed simultaneously by TGA-DSC Discovery of TA Instrument (New Castle, DE, USA). TGA was recorded for 50–500 °C at 10 °C/min under air atmosphere, while the DSC was run for 150–550 °C at 10 °C/min under nitrogen atmosphere, but the crucible lid was pierced seven times, allowing the gas to escape. SEM images were registered at high vacuum for secondary electrons with acceleration voltage of 1–5 kV, recorded by field-emission equipment JEOL JSM-7600F (Tokyo, Japan). The sample was dispersed by ultrasound bath in isopropyl alcohol.

The standard test of resistance to *Trametes versicolor* (CDBB-H-1051) followed the ASTM D-1413-07 test method for wood preservatives by laboratory soil-block cultures [16]. The test-blocks, made of composite, were 2 cm on each face and were oven-dried and maintained in a desiccator until fungi impregnation. In order to cast such a composite-block, the composite-powder was agglutinated by adding acryl-styren resin Joncryl 1522 from BASF (Ludwigshafen, Germany). For comparison, a second viny-acrylic resin, called QV from POLIMEROS ESPECIALES (Cuautitlán Izcalli, Mexico) was applied as a film to cover the test-block surface over selected test blocks. All test-blocks were sterilized. The Malt Agar substrate was prepared with 2% Malt extract and 1.5% Agar, while the blind test contained just water and 1.5% Agar. The soil culture bottles were prepared with a 10 mm square fungus inoculum section, aged for three weeks, then placed over the wood feeder strip and incubated until it was covered by mycelium. Then these culture bottles were ready to receive the test blocks. After 12 weeks in the incubation room, the calculation of weight loss of every test block was performed according to Equation (1).

(1) $$\frac{T_{1}-T_{2}}{T_{1}}\times 100$$

where *T*_1_ is the initial weight and *T*_2_ is the final weight.

The inoculum was prepared with 9 g of fungus mycelium, cultured in Malt–Agar and 81 mL water. The test-block was cultured with 1 mL of this solution and no further wood preservative was added.

## 3. Results and Discussion

The synthesis and characterization of the functionalized materials were followed by FTIR.

### 3.1. Fourier Transform Infrared Spectroscopy

**Figure 1:**
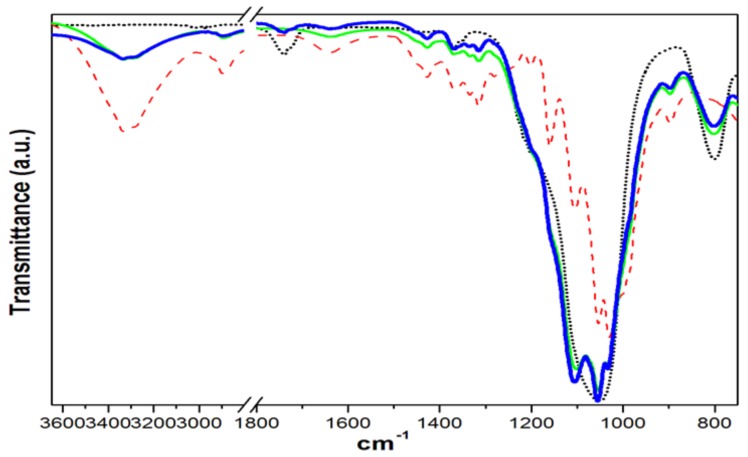
FTIR spectra of C20 (red dash line), NSi (black dot line), CP1 (green line), CP2 (blue line).

The most intense absorption peaks of C20, Figure 1, are found in 3342 cm^−1^ due to OH frequency region of intramolecular hydrogen bond vibration of secondary alcohol C3–OH⋯O5 jointly with the 3277 cm^−1^ peak due to the similar vibration of C2 secondary alcohol [17], and within the area of 1200–1000 cm^−1^ several absorption peaks are present due to C–O stretching vibrations from the glucose ring skeletal vibration [18].

The weak antisymmetric vibration of C–O–C is in 1161 cm^−1^ of the glycoside links, while the 1106 cm^−1^ vibration is probably due to the C–O group which belongs to the same secondary alcohol as the one at the origin of the OH stretch mode at 3277 cm^−1^. The most intense band is in 1033 cm^−1^ due to C–O group of C6H2–O6H primary alcohols in dominant conformation, while its secondary conformation is observed in 1000 cm^−1^. The next band in 1055 cm^−1^ is due also to C–O group of C3–O3H secondary alcohols [17].

The FTIR spectrum of NSi looks very different from cellulose, Figure 1, since it only shows a very intense band in 1052 cm^−1^, due to asymmetric mode of Si–O–Si correlated with the next intense band due to symmetric mode of the same Si–O–Si found in 800 cm^−1^ while the weak band in 1741 cm^−1^ is related to C=O group vibration since NSi is a modified silica [19].

**Figure 2:**
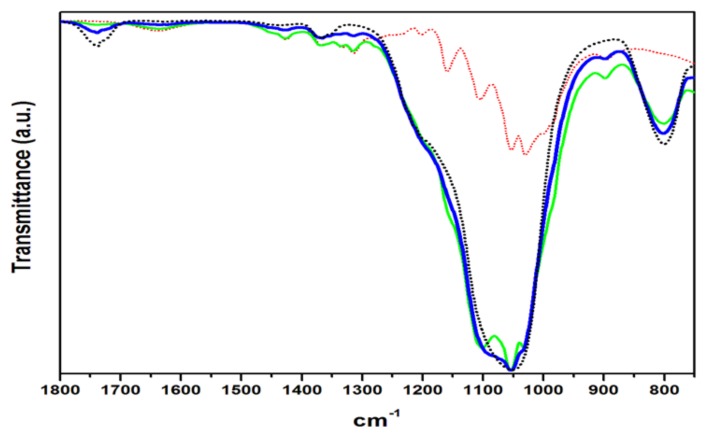
FTIR spectra of AG20 (red dot line), NSi (black dot line), CP3 (green line), CP4 (blue line).

On the other hand, the FTIR spectra of composites CP1 and CP2, Figure 1, clearly shows that the band in 1741 cm^−1^ is only present in CP2, the composite of equal proportions of cellulose and silica, indicating that the organic matrix is segregating it and it is accepting no more than 43 wt % of NSi to disperse homogeneously into the fibers of the composite CP1. Such behavior is also observed in Figure 2, showing the scale only from 1800–750 cm^−1^ for zooming the weakest bands, where the spectrum of CP4 shows the same band of 1741 cm^−1^, meaning that the surface modification of pure cellulose to get AG20 is not affecting the fibers capacity to receive and disperse higher amounts of inorganic particles from 43 to 50 wt %.

**Figure 3:**
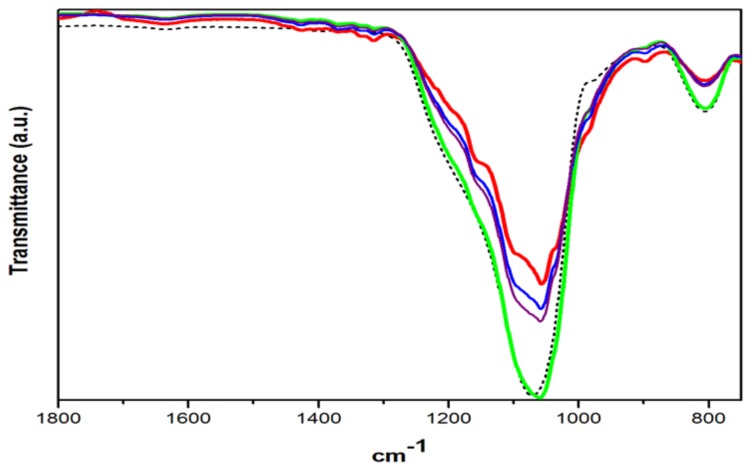
FTIR spectra of ASi (black dash line), CP19 (red line), CP20 (green line), CP21 (blue line) and CP22 (purple line).

Figure 3 shows the same scale as that of Figure 2, in order to compare the effect of surface modification by solvent exchange method in CP19 up to CP22, when the inorganic particle size is just 12 nm of ASi. Firstly, ASi shows a weak band in 978 cm^−1^, such band is absent in NSi. A study of fly ash activation reports an intense band in 989 cm^−1^ related to Si–O asymmetric stretching vibration, which explains that the lower wavenumber of this band was associated with a lower degree of crosslinking of the amorphous phase of silica [20]. The same weak band appears in the composites between 980 and 983 cm^−1^. The CP20 spectrum looks much more like ASi than CP19 but CP21 and CP22 spectra look much more like the cellulose matrix or like CP19, indicating that the pure cellulose composites accept the inorganic particles up to 43 wt %, while the cellulose matrix modified by solvent-exchange method, AG20, disperses up to 50 wt % even retaining the cellulose characteristic vibration bands in CP22. Such behavior has been observed in previous works of this working group [21,22]. The high amounts of inorganic nanoparticles and also the particle size dispersed into the AG20 matrix will affect also the thermal stability of the composites.

The weak band in 1635 cm^−1^ has been related to the fibers water absorption [23]; it was observed in C20, Figure 1, and even with less intensity in AG20, Figure 2, but it is practically not seen in Figure 3, thereby indicating that the fibers’ hydrophilic character was dramatically reduced, since hydroxyl groups exposition was hindered by the silica nanoparticles [24].

Some authors suggest that after surface modification of the fibers, the presence of peaks in the region near to 450, 800 and 1100 cm^−1^ indicate that chemical bonding between cellulose and silica nanoparticles is taking place, since the band in 1100 cm^−1^ is assigned to Si–O–C stretching vibration [25,26]. In this study, even when no precursors of the inorganic nanoparticles were used and no crosslinking agent or catalyst was used, the characteristic vibration bands of cellulose and silica were observed in these hybrid composites produced in a facile synthesis way.

### 3.2. Scanning Electron Microscopy

**Figure 4:**
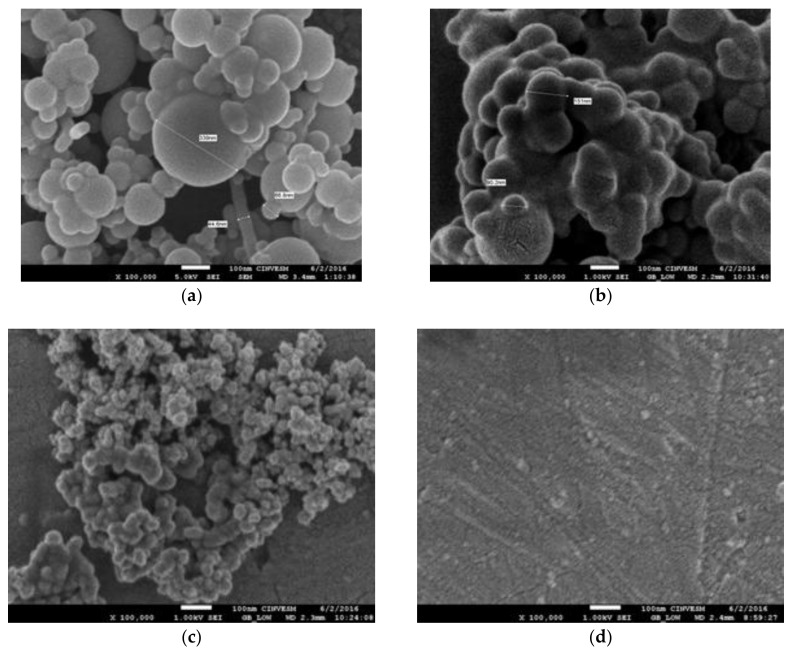
SEM micrographs of composites at 100,000×. (**a**) CP2 composite; (**b**) CP4 composite; (**c**) CP20 composite and (**d**) CP22 composite.

The dispersion of the reinforcers within the matrix C20 and AG20 in the composites was analyzed by scanning electron microscopy. The comparison of micrographs of CP2 vs. CP4 and CP20 vs. CP22 under 100,000 magnifications are shown in Figure 4. The micrograph of CP2 shows an aggregation of well delimited sphere shapes particles that are bigger than 300 nm while the CP4 micrograph shows, for the same magnification, a more continuous surface of such aggregation of sphere-shaped particles containing the biggest one of 150 nm. This comparison enabled us to consider that using the same quality of reinforcer, NSi, the composite resulting is affected by applying solvent exchange method to the pure matrix. Such a reaction could result in improved embedding of the filler within CP4 matrix, strengthened by the enhanced filler matrix interactions due to the matrix surface modification. However, the particle size distribution of every composite must be measured in the near future in order to clarify how this treatment is affecting the composites. The comparison of CP20 and CP22 focuses the attention on the effect of particle size since ASi is the filler that shows nearly one-tenth of NSi size. The micrograph of CP20 enabled us to see a more homogeneous distribution of particle size with values of 24 and 55 nm (not shown in picture) while the CP22 micrograph shows an even more continuous surface indicating that again the modification of the matrix has affected the distribution of filler giving place to a nice continuous morphology. In sum, we can say that using the filler of ASi (12 nm size) is very convenient for producing homogeneous composites when the matrix has been modified by a solvent exchange method such as AG20. Comparing the four composites micrographs allows us to see a progressive plasticizing effect that is clear in CP22 where the matrix is AG20 and the filler is ASi in 50 wt % content. This effect is very important to notice since no agglutinating resin was added in these composites.

Exactly the opposite effect has been observed in a study of cellulose acetate butyrate (CAB) and cellulose nanowhiskers (CNW) hybrids obtained with nearly the same method used by us. Under 25,000× the CAB looks neat and smooth on the contrary the nanocomposite containing 12 wt % of CNW under 50,000× shows voids and also dots considered as transversal sections of whiskers embedded in the matrix, the absence of aggregates at micrometer scale confirms the good dispersion of CNWs but no smooth surface or plasticized result is observed in these hybrid materials [27].

### 3.3. Thermogravimetric Analysis

**Figure 5:**
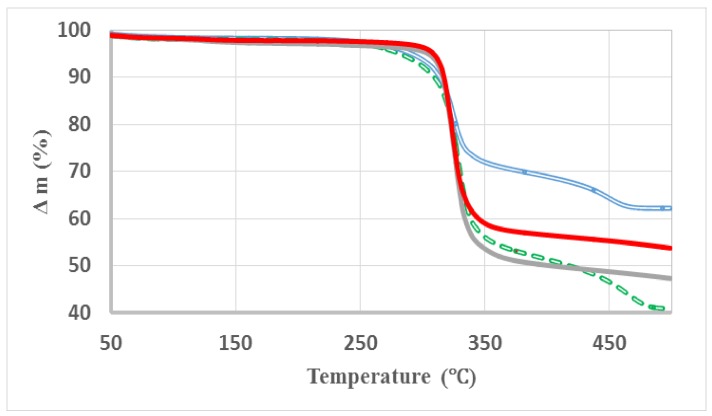
TGA curves of CP2 (green line), CP4 (blue line), CP20 (gray line) and CP22 (red line).

In order to determine the silica content in the composites, TGA measurement was carried out in air. Figure 5 shows the TGA curves of CP2, CP4, CP20 and CP22. In these samples, the thermal degradation occurs mainly at about 324 °C, but the most interesting point is the residual weight remaining at 500 °C. The composite CP20 of C20 matrix and ASi reinforcer contains residual weight of 47.3%, that is close to the nominal 50 wt % remanent according to the formulation indicating that this composite released 2.7 wt % more than expected. On the other hand, composite CP22 of AG20 matrix and ASi reinforcer contains residual weight of 53.7% indicating that this formulation retained 3.7 wt %, possibly due to the inorganic particles stabilization into the matrix fibers net. About composite CP2 of C20 matrix and NSi reinforcer according to the formulation 50 wt % of residual silica content must be expected, but this sample retains only 41% indicating again that 9% is released in excess, akin to CP20. In such C20 matrix samples, an excess of mass is released after 500 °C treatment. On the contrary, composite CP4 of AG20 matrix and NSi reinforcer releases only 38 wt % retaining 62 wt %, meaning that 12% more weight is retained in this composite in comparison with the calculated content of silica. An excess of weight is found in the composites of AG20 matrix after 500 °C treatment as in the case of CP22. An explanation of this large amount of residual mass in CP4 could relay in the thermal capability of NSi that can be shielding the composite matrix avoiding its complete thermal degradation due to the chemical functionalization of NSi that it is not the case for ASi.

The complete series of composites, 25, 50, 75 and 100 wt/wt, of C20 and AG20 matrix with the same reinforcer NSi was measured. The data comparison indicates that the residual mass amount even at 550 °C shows a trend from 20% up to 55% when 75% of reinforcer was added, but it differs significantly when the higher amount of filler is contained, 100%, meaning the 50% of composite total mass; when C20 is the matrix it delivers just 41% of residual mass while in AG20 matrix it is 62 wt %. Such a result suggests that the matrix treated by solvent exchange method is better stabilized by the reinforcer retaining more mass than the matrix that is just untreated.

Recent studies have been conducted of thermal degradation of pure cellulose microfibrils with no content of hemicelluloses, pectins and lignin informed by TGA and dTGA measurements under nitrogen gas the maximum of thermal degradation at 370 °C, but the complete thermal event starts at 250 finishing at 380 °C with 16 wt % of residual mass after 450 °C [28]. While a study by TGA and dTGA of the same cellulose powder used in this work under the same heating rate shows its maximum thermal degradation at 317 °C, but the complete thermal event takes also from 270 to 340 °C with a residual mass of 4 wt % after 420 °C [29].

About the thermal stability of cellulose/silica hybrid composites, created by a sol-gel method using TEOS precursor of silica, bleached pulp and tungstophosphoric acid (H_3_PW_12_O_40_) (EPTA). The thermal degradation of the organic material was observed at 305 °C and at 345–350 °C for the hybrid materials indicating that with 51% (wt/wt) of SiO_2_ content in the cellulosic composites clearly confers higher thermal resistance to those materials. This communication also relates the thermal conductivity coefficient of these materials with the effect of silica content showing its capacity as conventional insulation material [30].

When using bacterial cellulose (BC) hydrogel for preparing composites with TEOS, the BC hydrogel was immersed into 10 and 20% aqueous TEOS dispersions delivering silica deposited on BC microfibrils via silanol. The TGA measurements indicated that thermal degradation occurs at 320 °C and after 500 °C the silica content was 43 wt % of the hybrid composite prepared with 20% TEOS. Those TGA curves look very similar to those of CP2 and CP4 of this work [31].

Another group of data also supports the synergy effect of the synthetic process, that it generates materials with new properties and potential applications. When SiO_2_ nanoparticles were chemically bonded on the surface of the cellulose fibers the thermal stability of these fibers was improved. Even a reduction of up to 50% in the moisture adsorption capacity of the modified cellulose fibers was observed [24,32]. Although these study hybrid composites were produced by a very facile synthesis, its thermal behavior shows very competitive performance.

### 3.4. Differential Scanning Calorimetry

**Figure 6:**
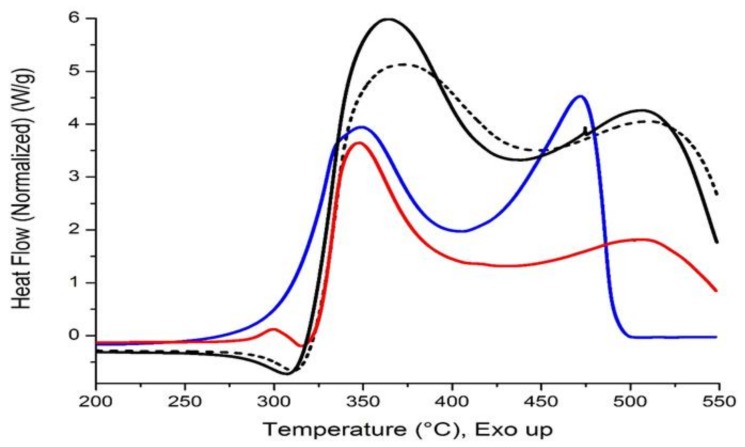
DSC curves of CP4 (blue line), CP22 (red line) versus AG20 (short dash line), C20 (black line).

The results found by DSC are quite interesting, since it is reported that cellulose pyrolysis shows a clear endothermic peak around 350 °C, but at temperatures higher than 400 °C exothermal properties of the related reactions in cellulose pyrolysis were observed [33]. In the present study, the effect of reinforce particle size on the same matrix, AG20, is analyzed on CP4 with NSi and CP22 with ASi, see Figure 6. The comparison of C20, AG20 against the composites shows a small endothermic peak around 310 °C: 306 in C20 and 311 in AG20 while in CP4 no endothermic peak was found but the degradation starts at 225 °C marking a clear difference with CP22 being the only composite that shows a small exothermic peak with maximum at 300 °C. When the temperature increased the next peak was exothermic, showing a clear shifting from 363 (C20) to 373 (AG20). CP22 and CP4 show that maximum in 347 and 349, respectively, but the last one also shows a shoulder found at lower temperature, 335 °C. The second exothermic peak shows a similar maximum among the composites and raw material: 505 (CP22), 507 (C20) and 511 (AG20), CP4 shows the maximum in 472 °C with the highest heat flow of all of them. In contrast, the composite with the lowest heat flow is CP22. The behavior of these composites is consistent with the mass loss observed by TGA measurements. Table 2 features the characteristic thermal events of selected composites and raw materials.

Among other working groups that have studied the degradation process of cellulose by DSC, one group found that degradation already starts at 220 °C, finishing at 475 °C with total mass loss of 97.6% [34]. They related the pyrolysis onset temperature by DTA with the cellulose crystallinity index citing the publication of Ciolacu and Popa, when the cellulose shows the lowest crystallinity index the degradation takes place at lowest temperature but the heating rate must be also considered [35]. The solvent exchange treatment brought better thermal stability to AG20 starting its thermal degradation 25 °C higher than pristine cellulose.

Another group that employed bleached pulp, TEOS and several catalysts to obtain hybrid materials by sol-gel method found, by TGA and DSC, that as the degree of the silica crosslinking increases the thermal stability of composites increases as well, even though the highest amount of silica incorporated was just 31.3% wt/wt. This explains that the endothermic peaks found in the region 310–340 °C correspond to segmental motions in the hybrid materials under degradation [36].

Considering the fact that the sol-gel method suggests covalent bonding between cellulose and silica in hybrids, we believe that no covalent bonding is present in our hybrid materials, since the thermal degradation starts with an exothermic peak in CP22, a rather electrostatic interaction takes place instead; however, dedicated work to clarify this effect is under progress.

### 3.5. Trametes Versicolor Resistance

**Figure 7:**
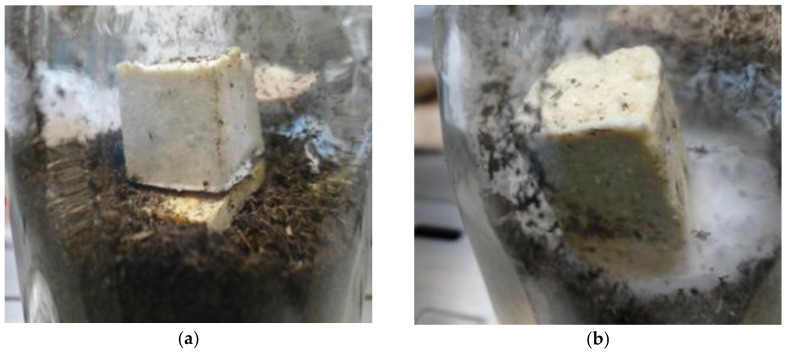
ASTM D-1413 standard test with *Trametes versicolor* of (**a**) CP22 and (**b**) CP22 with QV resin film.

The antifungal activities of the 4 samples of hybrid composites of CP22 composition were registered according to the ASTM D-1413 standard test method, just pure and with the film of QV 528 over some samples surface. Both images in Figure 7 show the comparison of *Trametes versicolor* growth on the composites cubes. The CP22 cube with no QV resin film coat shows no growth of fungi while CP22 with QV resin film shows just little overgrown by the test fungi. However, the weights record demonstrated that such QV resin is preserving the CP22 surface against the fungi attack since CP22 (a) shows a higher decrease of weight, 9%, while CP22 (b) decreased only 7.3 wt %. We must think about the chemical composition of each resin in order to explain this result. The acryl styrene resin (Joncryl) contains aromatic groups keeping the fungi growing at minimum since aromatics are toxic for the microorganism but the fungi can migrate into the composite bulk through the surface defects [37]. On the other hand, the vinyl acrylic resin (QV) contains aliphatic groups mainly favoring the fungi growth but when the resin film is adhered over the composites surface covering its defects it rejects humidity adsorption preventing the fungi attack. The growth inhibition of *Trametes versicolor* in these hybrid composites can be related also to its water absorption capability, lower water absorption means lower fungus growth capacity but no water absorption standard test was performed on these composites. The antifungal effect is on the same order that the decay resistance to the same fungus when untreated southern yellow pine particles were blended with 10% urea formaldehyde resin under the AWPA E10-91 standard test method for solid wood showing weight loss of just 8.1% of the particle board total weight after 12 weeks exposure [38].

A comparison of enzymolysis degradation by cellulase of sisal fibers and sisal self- reinforced composites has shown that under cellulose-to-specimen weight ratio of 1% after 10 h of reaction the composites are less susceptible to enzyme aided biodegradation than the fibers. The buffer solution of cellulase can only immigrate into the composite bulk through surface defects indicating low reaction probability [39].

The same standard test D1413 was applied to wood plastic composites (WPC), specimens prepared by hot press system with 40% polypropylene and 60% particles of pine, maple or oak wt/wt. After 12 weeks of *Trametes versicolor* exposure, the maple samples showed 16 wt % loss while the oak samples showed 13 wt % loss [40]. All these cases have shown lower resistance to *T. versicolor* than the composites prepared by our method.

On the other hand, these kinds of composites could be also used to protect paper artwork from deterioration of *Aspergillus versicolor* growth applying a thin film of composite to prevent the re-growth of fungi after cleaning the paper surface by microwave heating that has recently been reported by an Italian research group [41].

## 4. Conclusions

An environmentally friendly one-pot method was designed after the percolation approach for avoiding the grafting of molecules to the cellulose surface or the functionalization of the inorganic filler, silica nanoparticles. The key step of this work is using the solvent exchange strategy [42] for not drying the cellulose matrix, keeping the voids of biopolymer net to host the inorganic nanoparticles. The surface modification of cellulose by this method allowed the observation of its macroscopic hydrophobicity providing compatibility with the inorganic reinforcer up to 50 wt % registered by SEM images. Even when no contact angles of the samples were measured, we can say that the nanocomposite itself shows hydrophobic behavior hindering the fungal growth, thereby demonstrating high capability to use this material in exteriors such as building façades or as thermal insulator fillers, even when the material is not an aerogel.

This facile and cost-effective synthetic method prepares an uncommon kind of nanocomposite, since cellulose from several sources and varied types of particles and sizes are commonly used for reinforcing synthetic polymers [43] or other biopolymers such as starch, but it is not commonly used as matrix, except for all cellulose composites [44]. Also, the simplicity of the applied method opens up the possibility for application in high-volume processes of polymers that nowadays require the ability to withstand higher temperatures.

## Figures and Tables

**Table 1:** Composition and labelling of composites prepared from cellulose matrix.

Constituents wt %	C20	AG20	NSi	ASi
CP1	100	0	75	0
CP2	100	0	100	0
CP11	100	0	25	0
CP12	100	0	50	0
CP3	0	100	75	0
CP4	0	100	100	0
CP13	0	100	25	0
CP14	0	100	50	0
CP19	100	0	0	75
CP20	100	0	0	100
CP21	0	100	0	75
CP22	0	100	0	100

**Table 2:** Characteristic features of thermal decomposition of composites.

Sample	Tstart ^1^ (°C)	Tmax ^1^ (°C)	Tmax ^2^ (°C)	Tmax ^2^ (°C)	Mass Loss ^3^ (wt %)
CP2	225	324	350	485	59.1
CP4	223	327	349	472	37.9
CP20	280	325	350	505	52.7
CP22	290	324	347	505	46.3
AG20	250	325	373	511	97.0
C20	225	323	363	507	97.0

^1^ by dTGA, ^2^ by DSC exothermal peak, ^3^ up to 500 °C.
